# Disrupting Mosquito Reproduction and Parasite Development for Malaria Control

**DOI:** 10.1371/journal.ppat.1006060

**Published:** 2016-12-15

**Authors:** Lauren M. Childs, Francisco Y. Cai, Evdoxia G. Kakani, Sara N. Mitchell, Doug Paton, Paolo Gabrieli, Caroline O. Buckee, Flaminia Catteruccia

**Affiliations:** 1 Center for Communicable Disease Dynamics, Harvard T. H. Chan School of Public Health, Boston, Massachusetts, United States of America; 2 Department of Epidemiology, Harvard T. H. Chan School of Public Health, Boston, Massachusetts, United States of America; 3 Department of Immunology and Infectious Diseases, Harvard T. H. Chan School of Public Health, Boston, Massachusetts, United States of America; 4 Dipartimento di Medicina Sperimentale, Universita' di Perugia, Perugia, Italy; Stanford University, UNITED STATES

## Abstract

The control of mosquito populations with insecticide treated bed nets and indoor residual sprays remains the cornerstone of malaria reduction and elimination programs. In light of widespread insecticide resistance in mosquitoes, however, alternative strategies for reducing transmission by the mosquito vector are urgently needed, including the identification of safe compounds that affect vectorial capacity via mechanisms that differ from fast-acting insecticides. Here, we show that compounds targeting steroid hormone signaling disrupt multiple biological processes that are key to the ability of mosquitoes to transmit malaria. When an agonist of the steroid hormone 20-hydroxyecdysone (20E) is applied to *Anopheles gambiae* females, which are the dominant malaria mosquito vector in Sub Saharan Africa, it substantially shortens lifespan, prevents insemination and egg production, and significantly blocks *Plasmodium falciparum* development, three components that are crucial to malaria transmission. Modeling the impact of these effects on *Anopheles* population dynamics and *Plasmodium* transmission predicts that disrupting steroid hormone signaling using 20E agonists would affect malaria transmission to a similar extent as insecticides. Manipulating 20E pathways therefore provides a powerful new approach to tackle malaria transmission by the mosquito vector, particularly in areas affected by the spread of insecticide resistance.

## Author Summary

Mosquito control is the only intervention that can reduce malaria transmission from very high levels to close to zero. However, current mosquito control methods are severely threatened by the rapid spread of insecticide resistance in anopheline mosquito populations that transmit the malaria-causing *Plasmodium* parasites. Here we show that when steroid hormone signaling is interrupted in female *Anopheles* mosquitoes, various aspects of their lifecycle are disrupted—females produce and lay fewer eggs, do not mate successfully and die more rapidly. Furthermore, they become less likely to be infected by malaria parasites. When we model the impact of steroid hormone agonists on malaria transmission, we predict that these compounds would provide an important new tool against malaria, particularly in regions of widespread insecticide resistance.

## Introduction

Despite recent progress in combating the malaria parasite, nearly 200 million infections and around 500,000 deaths are caused by malaria annually, mostly in young children in sub-Saharan Africa [1, 2]. Even with new drugs and vaccines in the research pipeline [3], control of the *Anopheles* species that transmit human malaria remains the cornerstone of prevention and transmission reduction efforts [2, 4]. Of the four classes of insecticides available for malaria control, pyrethroids are the only compounds approved for use on long-lasting insecticide-impregnated bed nets (LLINs), due to their relatively low toxicity, and they are heavily used in indoor residual spray (IRS) programs [5]. This is a major limitation, as the increased application of both interventions over the last decade has inevitably led to the emergence and spread of insecticide resistance in natural mosquito populations. Indeed, resistance to pyrethroids has been observed in most *Anopheles* populations from sub-Saharan Africa [6], making the identification of alternative non-toxic compounds that can reduce parasite transmission a high priority in the malaria control agenda [1].

Insecticide-based interventions impact malaria transmission by increasing the mortality rate of exposed female mosquitoes and, in the case of LLINs, by preventing them from biting humans. Mathematical models developed to aid in the design of malaria elimination programs during the first global eradication campaign showed the importance of increasing mosquito mortality [7, 8], which reduces the probability that mosquitoes will survive for the 12–14 day incubation period of the malaria parasite [9]. However, other aspects of adult mosquito biology that determine vectorial capacity, such as host preferences for blood-feeding, susceptibility to parasite development, and reproductive fitness, have not yet been fully exploited for malaria control.

*Anopheles* population densities are driven by the complex mosquito lifecycle involving multiple gonotrophic cycles in fertilized females. Following a single insemination event, a female stores sperm for her lifetime, using it to fertilize each egg batch produced after a blood meal [10–12]. Many of the processes characterizing this reproductive cycle are regulated by 20-hydroxyecdysone (20E), a steroid hormone originally studied in insects for its fundamental role in larval molting [13]. Besides an essential function of female-produced 20E in triggering egg development after blood feeding [14–16], in *Anopheles gambiae*, as well as in other important anopheline vector species, sexual transfer of this hormone by the male induces a dramatic series of molecular events that culminate in increased oogenesis, induction of egg laying, and loss of the female’s susceptibility to further mating [17–20].

Based on its multiple physiological effects, it is reasonable to speculate that 20E signaling pathways in the female mosquito could be exploited to manipulate reproductive success and possibly other aspects of mosquito biology that are relevant for vectorial capacity. To this end, synthetic 20E non-steroidal agonists such as dibenzoylhydrazines (DBHs), which mimic the action of 20E by competitively binding to the ecdysteroid receptor, resulting in high ecdysteroid activity [21, 22], could be utilized. When provided to larvae of some Lepidopteran and Dipteran species, DBHs induce precocious and incomplete molting, ultimately leading to death [21–25]. These compounds have extremely low toxicity to mammals and are non-carcinogenic [26, 27], and although reduced fitness of adult stages following DBH exposure has been documented in agricultural lepidopteran species [24], their potential use against adult stages of malaria vectors has not been tested.

Here we show that topical application of the non-steroidal ecdysone agonist methoxyfenozide (a DBH compound, herein referred to as DBH) significantly limits the reproductive success of adult *An*. *gambiae* females and greatly increases their mortality. Furthermore, females exposed to DBH are significantly less susceptible to infection with the human malaria parasite *Plasmodium falciparum*. We incorporate our experimental findings into a mathematical model of the mosquito life cycle to determine the potential impact of these multiple biological effects on mosquito population dynamics and malaria transmission. Our results suggest that the application of compounds targeting ecdysteroid pathways on impregnated bed nets or in indoor spray programs would significantly reduce malaria transmission, achieving results comparable to those from the use of insecticides. Manipulating 20E signaling in *Anopheles* mosquitoes therefore provides a new strategy for malaria control, especially needed in areas of widespread resistance to insecticides.

## Results

### Disrupting steroid hormone signaling in *An*. *gambiae* females affects mosquito traits central to vectorial capacity

To determine if steroid hormone signaling could be disrupted to manipulate entomological parameters key to malaria transmission, we treated *An*. *gambiae* females with the 20E agonist DBH and assessed its effects on egg laying and mating success, two reproductive traits that can impact mosquito population size and hence the frequency of encounters with the human host. While 20E is essential for egg development in insects, it has been shown that levels above a critical threshold can induce apoptosis of ovarian follicles [28, 29]. We therefore reasoned that topical application of DBH to the female’s thorax might disrupt egg development and thus reduce the number of eggs laid. Moreover, as 20E injections in virgin females completely abolish insemination in a number of *Anopheles* species [20], we tested whether exposure to DBH might achieve the same result. We also expected an effect of DBH application on female mortality, as, in *Drosophila melanogaster*, 20E is known to regulate longevity [30, 31], a crucial parameter of malaria transmission.

**Fig 1:**
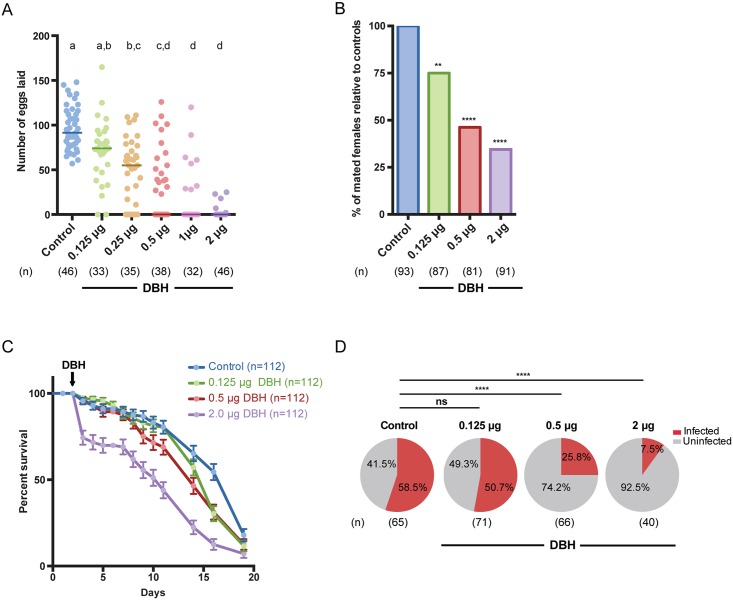
Disrupting steroid hormone signaling by topical application of the 20E agonist methoxyfenozide (DBH) affects oviposition, mating, longevity and *P*. *falciparum* development. **(A)** DBH-treated mated females failed to oviposit or laid a significantly lower number of eggs after a blood meal in a dose-response manner; whereas oviposition occurred in 100% of controls (Kruskal-Wallis test, *p* < 0.0001). Letters indicate post hoc significance (Dunn’s post hoc test). **(B)** The ability of virgin DBH-treated females to become inseminated was significantly reduced compared to control females (Fisher’s exact test for 2 μg, 0.5 μg, and 0.125 μg DBH: *p* < 0.001, *p* < 0.001, *p* = 0.024) **(C)** The median survival time of DBH-treated females was significantly lower than control-treated females (Log-rank test for 2 μg, 0.5 μg, and 0.125 μg DBH: *p* < 0.0001, *p* = 0.0044 and *p* = 0.0141, Median survival: 2 μg, 0.5 μg, and 0.125 μg DBH = 11,14, and 16d; Control = 19d). Data are presented as the percentage of survival of 4 replicates with Standard Error. Arrow indicates day of DBH application. (**D**) Females treated with 2 μg or 0.5 μg DBH showed an 87% and 56% reduction in *P*. *falciparum* infection prevalence (measured as number of females with oocysts 7 days post-infectious blood meal compared to controls), respectively (Fisher’s exact test for 2 μg and 0.5 μg DBH: *p* < 0.0001 and *p* < 0.0001). The number (n) of females analyzed is indicated in each panel.

Egg laying, mating, and lifespan were all significantly altered in females treated with this 20E agonist (Fig 1). We first tested mated females for their ability to develop and lay eggs when exposed to DBH 24h prior to blood feeding at 5 different doses (ranging in a 2-fold dilution series from 2 μg– 0.125 μg per mosquito, respectively). While 100% of the control females oviposited after a blood meal, females exposed to DBH showed a dose-dependent reduction in oviposition, with 47.4% of individuals laying eggs at the intermediate dose (0.5 μg) and only 10.9% at the highest dose (2 μg) (Fig 1A). An ED_50_ dose of 0.5 μg (0.08–0.13 95% CI) was determined from the dose-response curve (slope: 2.32, R = 0.993). Moreover, even in cases where females oviposited, we detected at the three highest doses a significant reduction in the number of eggs laid, with a median egg number of zero compared to a median of 91.5 eggs in the control group (Fig 1A). Upon dissection of individuals in all DBH-exposed groups, we found that 98.7% of females had no follicular growth or yolk deposition in the ovaries despite having fully engorged on blood, demonstrating that the reduction in oviposition rates was due to impaired oogenesis. We did not observe induction of autogenous egg development after exposure contrary to what is reported in culicine mosquitoes after DBH treatment in larvae [32]. Microscopic analysis of ovaries after terminal deoxynucleotidyl transferase dUTP nick end labeling (TUNEL) assays detected extensive fragmentation of chromatin, indicative of apoptosis, limited to the primary ovarian follicles of treated females 24h after DBH exposure, while control females had no observable apoptosis and had otherwise normal follicular morphology (S1 Fig). Similar apoptotic follicles were observed after 20E injections, showing that the phenotype induced by DBH application recapitulates the effects induced by 20E (S1 Fig). It is worth noting that previous experiments where 20E injections induced egg laying [19] were performed in blood fed females that had completed egg development.

Mating and female longevity were also significantly reduced in a dose-dependent manner compared to controls as a result of DBH exposure (Fig 1B and 1C). After treating virgin females with 3 DBH doses (0.125 μg (low), 0.5 μg (intermediate), and 2 μg (high), corresponding approximately to the ED_10_, ED_50_ and ED_90_ from the oviposition data) and placing them with males for 2 days, we observed a 25–65% reduction in insemination rates, as determined by the presence of sperm in the spermatheca (Fig 1B). Moreover, DBH-treated females showed an eight-day reduction in median survival time at the highest dose compared to controls (median survival time in 2 μg DBH: 11 days; Control: 19 days) (Fig 1C), and lifespan was reduced even at the lowest dose (median survival time in 0.125 μg DBH: 16 days). Overall these results demonstrate a strong, dose-dependent effect of DBH on important determinants of vectorial capacity.

### DBH strongly impairs development of *P*. *falciparum* parasites in *An*. *gambiae* females

As 20E signaling is an important modulator of the female’s post-blood feeding physiology, we determined whether manipulating steroid hormone pathways via topical application of DBH also impacted the establishment of *P*. *falciparum* infection in the mosquito vector, using the three doses utilized in the mating and longevity assays. *P*. *falciparum* prevalence (NF54 strain) was significantly reduced 7 days post-infectious blood meal at the two higher DBH doses relative to the control. At the highest dose, only 7.5% of females who fully engorged on an infectious blood meal were positive for oocysts, corresponding to an 87% reduction in infection prevalence relative to controls (Fig 1D). When using the intermediate dose, 25.8% of females failed to develop an infection, providing a 56% reduction in prevalence. Limited effects were observed in the low dose treatment group, where oocyst prevalence (50.7%) was similar to the control group (58.5%). In those females that developed oocysts, the intensity of infection was not significantly affected (S2 Fig).

### Modeling the impact of DBH on mosquito populations predicts a shift towards younger females

**Fig 2:**
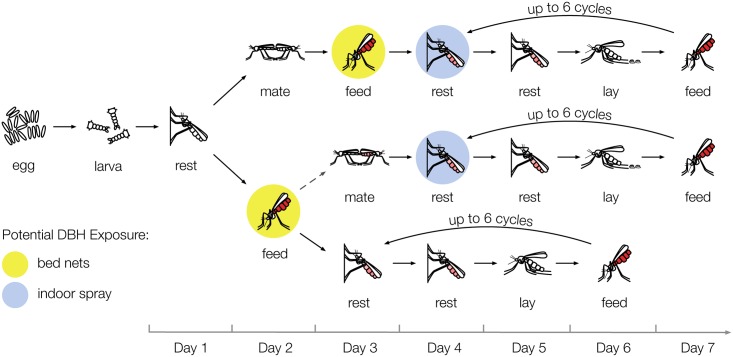
Schematic of mosquito life cycle model. Mosquitoes progress through the egg, larval, and pupal stages. Upon emergence, all mosquitoes rest outdoors for one day before mating first (50%) or feeding as virgins (50%). Some virgin feeders may subsequently mate, or, if exposed to and affected by DBH, may never mate. Afterwards, regardless of mating, all mosquitoes participate in up to 6 gonotrophic cycles, each cycle consisting of feeding (one day), resting indoors (two days), and ovipositing (one day). In our model, we assume that mosquitoes are exposed to DBH or insecticide only during their first feed (yellow circle) if applied via bed nets or only during their first indoor rest (blue circles) if applied via indoor spraying, and that mosquitoes exposed to DBH or insecticide via bed nets are nonetheless able to feed. Note that for indoor spraying, the bottom-most compartments are not used since in our model exposure always occurs after all mosquitoes have mated. In all gonotrophic cycles, DBH's effect on egg development reduces the number of eggs laid (no eggs are laid in the case of virgin mosquitoes). DBH also changes the age-dependent mortality starting on the day of first exposure and lasting throughout the mosquito's life. Insecticide increases mortality as well, but only on the day of exposure.

We developed a discrete-time deterministic mathematical model of the mosquito life cycle (Fig 2, S1 Table) to predict the effect of manipulating steroid hormone signaling via DBH on *Anopheles* population dynamics. We modeled mosquitoes through their life cycle, starting with juvenile aquatic stages (eggs, larvae, and pupae), followed by mating and up to six gonotrophic cycles consisting of blood feeding, resting, and ovipositing. We used our experimental findings to define the efficacy of DBH in our model (S1 and S2 Tables, Methods). Based on our experimental findings, at the highest dose the efficacy was defined to be a 95% reduction in egg batch size, 65% reduction in mating success, 87% reduction in *Plasmodium* infection risk, and an enhanced age-dependent mortality based on the experimental survival curves, with an 8-day reduction in median survival time (Fig 1, S2 Table). The efficacies defined for application of lower DBH doses can be found in S1 Table. Note that the purpose of this modeling approach was to elucidate the qualitative impacts of the multiple effects of DBH on the non-linear mosquito lifecycle, rather than to make quantitative predictions about its impact in field conditions.

We explicitly modeled the impact of DBH either delivered on impregnated bed nets to target females as they attempt to blood feed, or in indoor sprays to target females as they rest after feeding. Since our experimental results provide insight into a single exposure to DBH, and in order to compare with insecticide efficacy, we took a conservative approach and only considered possible exposure to DBH or insecticide to occur once, on the first feeding day in the case of treated bed nets or on the first indoor resting day for IRS. The insecticide was modeled at 100%, 80%, and 60% efficacy to reflect a situation of partial resistance emerging in the mosquito population [33]. In addition, we examined varying levels of coverage, i.e. the proportion of mosquitoes exposed in their first feed or first day of indoor rest (S3A Fig, Methods). To qualitatively examine the relative efficacy and general mechanisms of transmission reduction following a DBH-based intervention, we used a single well-mixed mosquito population without spatial structure (Methods).

**Fig 3:**
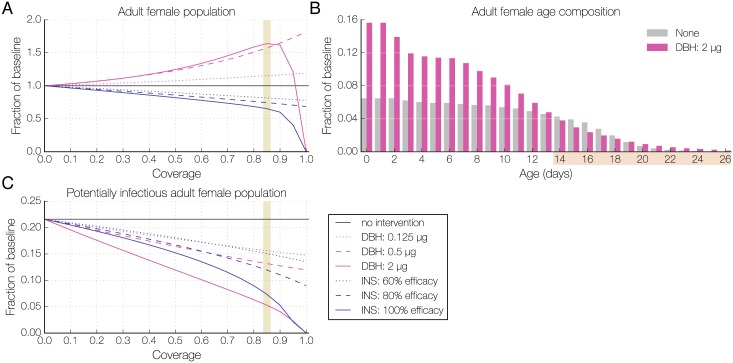
Effect of bed net-based interventions on the adult female mosquito population and individual age classes. **(A)** The adult female mosquito population varies non-linearly under increasing coverage with different DBH doses (pink lines) or insecticide efficacy (blue lines). The vertical yellow bar indicates 85% coverage, which results in the largest female adult population and for which the age composition of the population is considered in (B). The population size shown is relative to the total female population in the absence of any interventions (black line). **(B)** The age composition of female mosquitoes in the presence of 2.0 μg DBH (pink) or the absence of any intervention (gray) at 85% coverage, indicated by a yellow bar in (A) and (C). The highlighted days in the x-axis indicate the age range of mosquitoes that are old enough to transmit malaria if infected. **(C)** The potentially infectious adult mosquito population under increasing levels of coverage with different DBH doses (pink lines) or insecticide efficacy (blue lines). This includes females at least 12 days after a blood meal, and in the case of DBH exposure, a proportion of these females are excluded due to reduced *Plasmodium* susceptibility. The yellow bar indicates 85% coverage, for which the age composition of the population is considered in (B). Without intervention, the proportion of mosquitoes at least 12 days after their first feed (black line) is 0.22. Insecticide of 60%, 80%, or 100% efficacy and DBH of experimentally determined efficacy (Fig 1) are used.

The modeled mosquito population and its age structure were significantly altered by the use of DBH in bed nets and indoor residual sprays (Fig 3, S4 Fig). The relationship between DBH exposure and mosquito population size was non-linear. At the strongest dose and highest levels of coverage the mosquito population was driven to extinction, but for most levels of coverage the total adult mosquito population actually increased with DBH exposure due to reduced density-dependent larval mortality (Fig 3A, S5A Fig), consistent with previous models of vector control interventions [34–36]. However, this effect was accompanied by a shift in the age distribution of adult females towards younger individuals (Fig 3B). At the low and high DBH doses, for all levels of coverage, this shift alone reduced the proportion of females that lived long enough to transmit malaria, i.e. females surviving at least 12 days following their first blood meal (S5B Fig). Since our experimental results show that DBH also blocks *P*. *falciparum* infection, we combined this shift in age structure with the dose-dependent reduction in susceptibility to estimate the overall impact on the fraction of potentially infectious mosquitoes (Fig 3C). In comparison to insecticide exposure at the same coverage, low and intermediate doses of DBH showed a similar reduction in adult mosquitoes able to transmit malaria to that of 60% and 80% insecticide efficacy, respectively, while high DBH dose performed comparably if not better than a 100% effective insecticide (Fig 3C). Delivery of DBH through indoor spraying showed similar effects on the mosquito population (S4 Fig).

### Targeting steroid hormone signaling is predicted to substantially reduce malaria transmission

To examine the impact of DBH on malaria, we extended our mathematical framework to include malaria transmission with feedback between infectious human and mosquito populations (S6 Fig; S1 Table). In the model, mosquitoes required at least 12 days following an infectious blood meal to become infectious to humans [9]. Given the non-linear relationships between components of the model, transmission intensity prior to interventions may influence their relative impacts. We therefore considered three transmission settings, with high (85%), moderate (45%), and low (5%) malaria prevalence pre-intervention, and different DBH doses (2 μg, 0.5 μg, and 0.125 μg). Importantly, our results are not intended to quantitatively reproduce field conditions, but rather to reflect the relative reductions for a range of pre-intervention transmission settings and in comparison to the use of insecticides. As mentioned above, for simplicity we consider the effectiveness of each intervention to be the reduction in malaria prevalence after a single exposure relative to the pre-intervention prevalence, and in our model mosquitoes become exposed to DBH or insecticides only at the time of their first blood feeding (or their first day of indoor rest for IRS).

**Fig 4:**
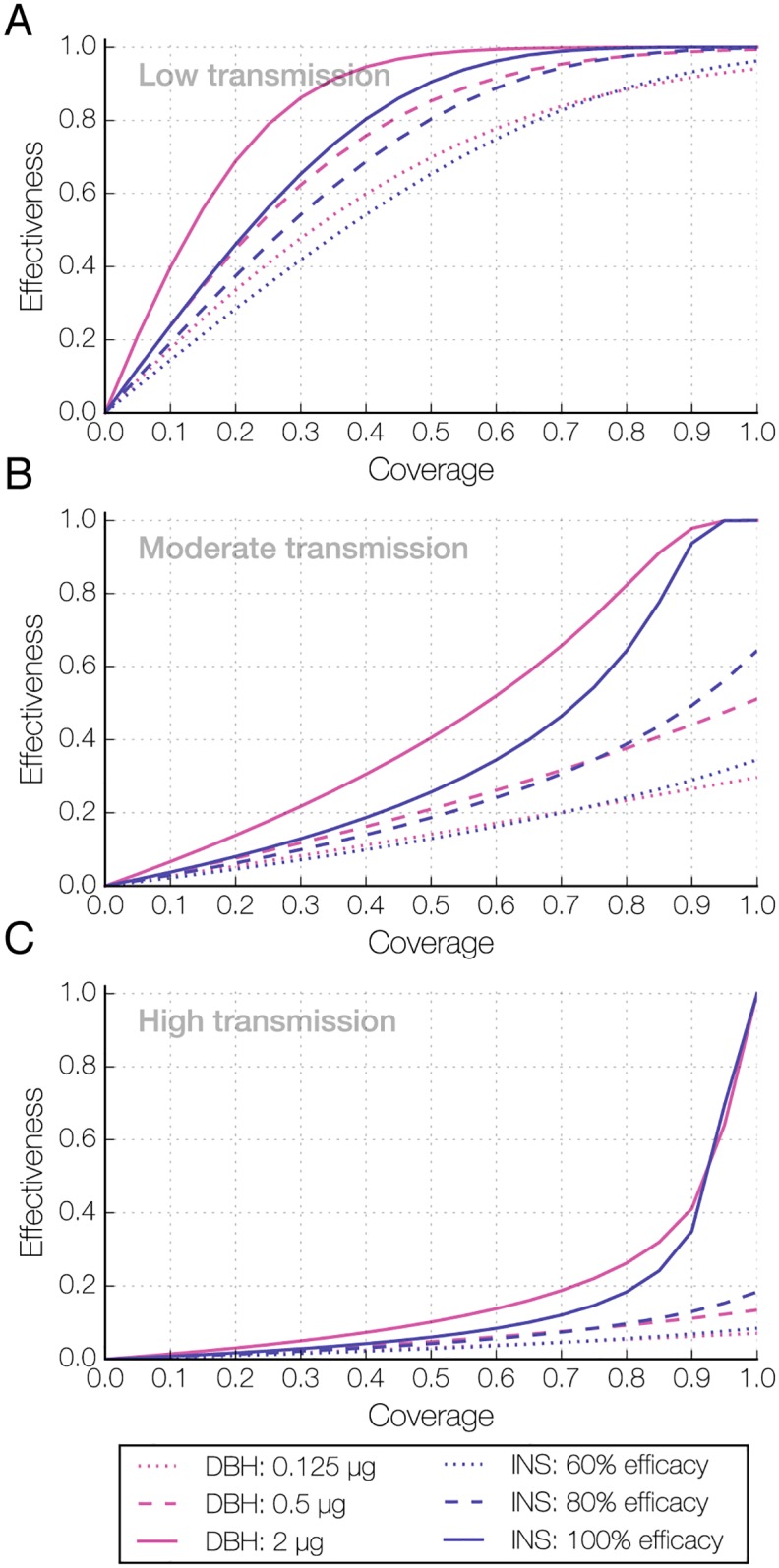
Effect of interventions on malaria under varying coverage. Effectiveness against malaria considering changes in both coverage (x-axis) and efficacy (line style). In low **(A),** moderate **(B),** and high **(C)** transmission settings, the effectiveness (reduction in malaria relative to pre-intervention prevalence) of both DBH and insecticides increases as efficacy and coverage increase. DBH applied at 2 μg (solid pink line) had effectiveness greater than or comparable to 100% insecticide efficacy (solid blue line), while 0.5 μg (dashed pink line) and 0.125 μg DBH (dotted pink line) had effectiveness similar to that of 80% (dashed blue line) and 60% (dotted blue line) insecticide efficacy, respectively. The maximal DBH efficacy considered is our experimentally determined effects on egg development, mating, mortality, and *Plasmodium* susceptibility (Fig 1).

Application of 2 μg DBH via bed nets showed strong effectiveness against malaria prevalence, outcompeting the impact of any insecticide efficacy in all transmission settings (Fig 4). The effects of low and intermediate DBH doses were comparable to those of 60% and 80% insecticide efficacy respectively, regardless of initial malaria prevalence. Effectiveness increased significantly with increasing coverage for all doses of DBH. Similar dynamics were observed when modeling DBH use in indoor sprays (S7 Fig). Regardless of the method of application or dose considered, a single exposure to DBH led to a reduction in malaria prevalence in all transmission settings at all coverage levels. We therefore expect that repeated exposures over the course of a female’s lifespan would have a larger impact on malaria.

## Discussion

The development of non-toxic compounds that target the mosquito vector in novel ways will be essential for achieving malaria elimination goals [1]. Our study identifies steroid hormone signaling as a promising new target that can provide an effective and complementary approach to existing tools for vector control. Although our data are based on topical application, and therefore cannot directly be extrapolated to effectiveness in field settings, we observed a robust, dose-dependent impact of the steroid hormone agonist DBH on egg development, insemination rates, and adult female longevity in experimental applications. Moreover, DBH strongly prevented the development of the deadliest human malaria parasite, *P*. *falciparum*, a highly desirable feature for any compound used in malaria control strategies. While the effects on fecundity, insemination rates, and longevity are in agreement with previous studies linking high 20E activity to apoptosis of ovarian follicles [28, 29], reduced mating receptivity [19] and premature aging [30, 31], the observed reduction in *P*. *falciparum* infections was completely unexpected. In future studies it will be important to test steroid hormone agonists under field conditions, using different mosquito strains and parasite isolates, and to determine whether the effects on oogenesis and parasite development are linked, for example, via the induction of immune pathways.

Interestingly, recent studies targeting another insect hormone, the sesquiterpene juvenile hormone, have shown life shortening and sterilizing activity against adult *Anopheles* species [37–40]. Pyriproxyfen (PPF, a juvenile hormone analog) is currently available in LLINs for its combined efficacy with insecticides [37, 41, 42] although, to our knowledge, the effects of juvenile hormone agonism on *Plasmodium* development within the mosquito have not been tested. Upon optimization and testing of DBH or other chemistries targeting steroidal pathways for tarsal uptake, alternating the use of steroid hormone and juvenile hormone agonists in combination with insecticides may provide the key to preventing the spread of resistance to conventional insecticides and extending their effectiveness.

In our experimental setting, DBH had biological efficacy by topical application at higher but still comparable doses to permethrin in mosquitoes exhibiting pyrethroid target-site resistance [43], which is an important factor when considering both the cost effectiveness and safety of using these substances in the fields. We explicitly considered only the acute lethality of insecticide exposure in our model as the overall toll that insecticide resistance and sub-lethal doses of insecticide may impose on mosquitoes and parasites is only now being investigated [44]. As the incidence and severity of resistance increases in wild populations, understanding the biological effects of sub-lethal insecticide exposure will be increasingly important.

Our modeling approach illustrates how the different actions of steroid hormone agonists interact in non-intuitive ways to change mosquito populations and malaria prevalence in comparison to insecticides. For simplicity we measured effectiveness after a single DBH exposure, and for consistency in our comparison we modeled contact with insecticide only during the first blood feeding cycle. Thus our model results provide a conservative estimate of the possible outcomes of DBH and insecticide exposure. Despite this, they clearly indicate that steroid hormone agonists would perform comparably to insecticides in all malaria scenarios tested. Even at lower doses, when the individual effects on reproduction, longevity, and parasite development become less striking, the combination of these effects is powerful enough to achieve a substantial reduction in malaria, mostly due to a decrease in the potentially infectious adult female population.

As observed in other modeling studies, the release of density-dependent mortality plays an important role in the predicted impact of interventions [34–36]. For generalizability, we did not include fluctuations in carrying capacity, although variations in climatic conditions (e.g. rainfall, temperature, humidity) that determine the availability and quality of larval breeding sites are clearly important factors in the field that prevent populations from reaching equilibrium [45–48]. It is therefore likely that our framework is additionally conservative in that it over-estimates the increases in mosquito population size following DBH application. The counter-intuitive finding that malaria prevalence can be reduced even when mosquito population size increases highlights the importance of qualitative models of this kind that can identify potential multifactorial and non-linear effects of interventions. It will be important to validate the model and test the arising hypotheses in the field in future studies, since our results are qualitative and mechanistic, rather than providing quantitative predictions.

In addition to the physiological effects we have examined here, compounds belonging to the DBH class have additional characteristics that make them promising tools for malaria vector control. DBHs are not toxic to mammals (LD_50_ > 5000 mg/kg by ingestion for methoxyfenozide, which compares favorably to the LD_50_ = 430 mg/kg by ingestion of the pyrethroid permethrin commonly used on LLINs) and would therefore be ideally suitable for bed net-based strategies, where low toxicity is essential. Moreover, although resistance to DBH has been experimentally induced in larvae from a number of insect agricultural pests and has been described in wild populations with varying reports on the possible biochemical and genetic basis [24, 49–51], it has rapidly reversed when the intervention was withdrawn [24, 50]. Although resistance to DBH or any other active compound will inevitably occur at some point, different strategies such as rotation, mosaics, or combination with insecticide could be utilized to delay its emergence. Interestingly, interventions like DBH that have a combination of physiological effects and/or alter mosquito age structure, rather than causing instant lethality, may exert less selection pressure on targeted mosquitoes relative to conventional insecticidal approaches, whilst impacting malaria transmission to a similar degree. However, *Plasmodium* parasites could develop independent mechanisms of resistance against the as yet uncharacterized anti-parasitic activity exerted by DBH. In this event, this could potentially uncouple the DBH effects on mosquito physiology from those on parasite development. Based on the conserved role of 20E pathways in regulating female physiology in multiple anopheline species [20], we expect that compounds interfering with steroid signaling will be biologically effective against other important malaria vectors such as *An*. *arabiensis*, *An*. *funestus*, and *An*. *stephensi*. Approaches targeting these hormonal pathways could therefore be a potent addition to the limited toolbox of vector interventions for successful malaria control in Africa and other regions of the world affected by this disease.

## Methods

### Experimental methods

#### Mosquito rearing

*An*. *gambiae* mosquitoes of the G3 strain were employed in all experiments. All mosquitoes were maintained under standard insectary conditions (26–28°C, 65–80% relative humidity, 12:12 hours light/dark photoperiod). All stages of larvae were fed on a liquid suspension of powdered fish food and carp fish pellets (Tetramin, Tetra Co., Melle, Germany). As the larvae developed, growth trays were periodically split into additional trays to maintain optimum growth density and to refresh the medium. Upon pupation, pupae were transferred in plastic cages (BUGDORM, MegaView Science Co., Taiwan). If virgin mosquitoes were required for experiments, then pupae were separated by gender by microscopic examination at this point. Adult mosquitoes were provided with water and 10% w/v glucose *ad libitum*.

#### DBH compound

An 8% stock solution of the DBH methoxyfenozide (Sigma-Aldrich, 32507) was prepared in DMSO and from this concentration five serial dilutions (0.4%, 0.2%, 0.1%, 0.05% and 0.025%) were prepared in acetone, with a final DMSO concentration of 5%.

#### DBH bioassays

The DBH serial dilutions (0.5 μl) and control solution (5% DMSO in acetone) were topically applied to the thorax of *An*. *gambiae* females anesthetized on ice using a micropipette.

To assess oviposition rates and number of eggs laid, four-day old mated females (confirmed *post hoc* via the presence of sperm in the spermatheca) were treated with 5 DBH doses described above or DMSO-acetone as control in two biological replicates and 24h later were fed on human blood using a Hemotek membrane feeding system. Females were blood fed 24h after application as 20E strongly inhibits biting behavior [52]. Females were put into individual cups to lay eggs 2 days later and the total number of eggs oviposited or developed was counted after three nights. For the mating assays, four-day old DBH-treated and control virgin females were added into separate cages containing a large excess of 5 day-old virgin males, 24h following topical application. Forty-eight hours later successfully inseminated (mated) females from each cage were determined via dissection and the presence of sperm in the spermatheca in two biological replicates. For the longevity assays, two-day old virgin females treated with DBH doses (2 μg, 0.5 μg, and 0.125 μg per mosquito) or control solution were maintained in cups and supplied ad libitum with a 10% sucrose solution and water, in four biological replicates using a total number of 112 females per dose. Mortality rates after DBH or DMSO:acetone exposure were recorded daily for 19 days, encompassing the period needed by mosquitoes to become infectious.

#### *P*. *falciparum* infections

Two-day old virgin *An*. *gambiae* females were treated with three doses of DBH (2 μg, 0.5 μg, and 0.125 μg per mosquito) or control solution. Twenty-four hours after exposure treated females were transferred to a sealed, secure feeding/infection box and allowed to feed on an *in vitro* culture of mature stage IV and V *P*. *falciparum* NF54 gametocytes [53] provided through a heated membrane feeder for 30 min. Mosquitoes that failed to engorge fully were vacuum aspirated out of their containers directly into 80% ethanol and discarded. Seven days post blood feeding, females were vacuum aspirated into 80% ethanol and after 5 minutes, transferred out of the secure feeding box into PBS. Midguts were dissected out in PBS and stained with 2mM mercurochrome for 12 minutes. After staining, midguts were mounted on glass microscope slides in 0.2 mM mercurochrome in PBS, and oocyst prevalence and intensity were determined by examination at 400x on a compound light microscope. In order to account for increased mortality at higher DBH doses, 50–100 females (depending on DBH dose) were exposed per treatment group per replicate. Four replicates were carried out in total.

#### Microscopic examination and TUNEL

Two-day old virgin female *An*. *gambiae* were injected with 138nl of a 38mM solution of 20E (2.5 μg per mosquito) in 10% ethanol (described previously [19]) or 10% ethanol as a vehicle control. Similarly, two further groups of females were exposed to either 2 μg DBH in 5% DMSO/Acetone or the vehicle control. Twenty-four hours post treatment, females were knocked down on ice and their ovaries dissected out in PBS. Isolated ovaries were fixed for 40 minutes in methanol-free, 4% paraformaldehyde in PBS. Fragmented chromatin was labeled by TUNEL with the APOPTAG Fluorescein *in situ* apoptosis detection kit (EMD Millipore, Darmstadt, Germany) using a modified protocol. After labeling, tissues were transferred to a droplet of VectaShield mounting medium with DAPI (Vector Laboratories, Burlingame, CA, USA), and examined at 63x magnification on an Axio Observer Z.1 inverted compound microscope (Carl Zeiss AG, Oberkochen, Germany).

### Mathematical modeling methods

#### *Anopheles* population dynamic model

We developed a discrete-time deterministic mathematical model of mosquito population dynamics to examine the impact of a single exposure of DBH or insecticide on *Anopheles* mosquitoes (Fig 2). We chose to look at a single exposure to be consistent with our experimental effects following a single application of DBH. Parameter values for the different effects of DBH (egg development, mating, mortality, and *Plasmodium* susceptibility) were determined from our experimental results (in Fig 1), as described in “Estimation of DBH efficacy from data”, while other parameter values (S1 Table) were taken from the literature or are discussed below. To estimate the impact of DBH on malaria, we include malaria transmission and feedback between human and mosquito populations (S5 Fig). We have intentionally used a well-mixed, simple model in order to examine the relative efficacy of interventions, and we assume all blood meals occur indoors. In the field, we expect spatial heterogeneities, the multitude of mosquito species, and different biting behaviors to play an important role in determining the precise outcome of control programs. In particular, we expect the estimates to be least relevant when infected mosquitoes are rare, such as when approaching malaria elimination, as this breaks the standard mean field assumption.

In our model of the *An*. *gambiae* lifecycle (Fig 2), adult mosquitoes lay eggs, which spend three days as eggs [54] and ten days as larvae [55], a period combining both the morphological larval and pupal stages. Only female adult mosquitoes are modeled although male eggs and larvae remain to appropriately account for density dependence during the larval stage. Upon emerging as adults, females rest outdoors before either mating or feeding, and the 50% who feed as virgins [56] but do not encounter or are not affected by DBH, subsequently mate. We assume an excess of males such that every female can successfully mate. Upon mating, females enter repeated gonotrophic cycles consisting of: (i) feeding, (ii) resting indoors for two days to promote blood digestion and egg development and (iii) egg laying. Each activity takes place in the evening and mosquitoes engage in a single activity per day, the time step in the model.

A proportion of mosquitoes are lost to mortality each day, at rates that differ for eggs, larvae, and adults. The daily mortality rate for eggs assumes that approximately half (~51%) of eggs hatch after 3 days, below the estimated hatch rate in controlled laboratory conditions [47] to account for predation. While the daily mortality rate for eggs is fixed (d_e_ = 0.2) and age-dependent for adults (see below), we incorporate density dependence in larval mortality as a sigmoidal increase with the larval population size (S3D Fig):

d˜l(nl)=dlmin−c1+dlmax−(dlmin−c1)1+e−c2(nlK)+c3

where d_l_^min^ = 0.035 [57] is the baseline daily larval mortality rate, d_l_^max^ = 0.4 is the maximum daily larval morality [57], n_l_ is the total number of larvae, K = 10^4^ is the equilibrium larval population size under no intervention, and the shape of the function is determined by the constants: c_1_ = 0.05 to begin at the baseline daily mortality rate; c_2_ = 3.4 to control steepness of the curve, and c_3_ = 2 to control position of the curve, were chosen so the mortality rate at a population size of K results in exact replacement. Thus, we observe a stable mosquito population given our lifecycle parameters in the absence of intervention. Sensitivity analysis of the equilibrium larval population size under no intervention, K, showed nearly identical simulation results across parameter values spanning several orders of magnitude, of which we chose an intermediate value. Separate age-dependent adult mortality curves were estimated from the longevity bioassays for control and DBH-exposed mosquitoes. After six gonotrophic cycles at the baseline mortality rate [10], the proportion of the mosquito population remaining is small and is removed, yielding a maximum lifespan of 27 days.

The mating effect prevents virgin mosquitoes from mating with probability h_m_, although exposed virgin feeders, which cannot produce viable eggs, still enter gonotrophic cycles, potentially contributing to the spread of malaria through biting behavior. The egg effect scales the egg batch size by h_e_ between 0 and 1. The mortality effect is modeled by having separate age-dependent daily survival probabilities for DBH-unexposed and -exposed mosquitoes, with exposed mosquitoes experiencing higher risk of mortality earlier in life but lower mortalities after day 16 (S3B and S3C Fig, S2 Table).

Similar to DBH application, insecticide exposure occurs while feeding to simulate bed nets, and while resting to simulate IRS, and results in a proportion, d_c_, of mosquitoes killed, corresponding to an insecticide efficacy of 100%, 80%, or 60%, values chosen to reflect the development of partial insecticide resistance in the population [33]. Mosquitoes that survive have no lasting effects. Age-dependent survival, discussed earlier, and survival following insecticide exposure are assumed to be independent, so the product of their probabilities determines the overall daily survival probability.

We examined varying levels of coverage, i.e. the fraction of mosquitoes exposed in their first feed in the case of bed nets or first indoor resting cycle in the case of IRS (S3A Fig). Coverage is not equivalent to the fraction of mosquitoes exposed to an intervention, as even at 100% coverage, newly emerged mosquitoes that rest outdoors or mate prior to their first feed remain unexposed (Fig 2) and at higher coverage, exposed mosquitoes become increasingly underrepresented in the adult population since they have higher mortality as compared with unexposed mosquitoes. Since DBH, unlike insecticide, is modeled as having life-long effects, the assumption of a single exposure greatly reduces the number of compartments needed and affords computational simplicity, but underestimates the efficacy of intervention. In the case of bed nets, we assume mosquitoes that are exposed to DBH or insecticide still successfully feed on the day of exposure.

#### Incorporating malaria transmission

To examine the impact of DBH on malaria transmission, we added susceptible and infectious human populations to our mosquito lifecycle model (S5 Fig). The infectious human proportion: I_H_(t + 1) = β_h_(t)S_H_(t) − *ν*I_H_(t), depends on β_h_(t) = 1 − (1 − b)^*N* × *f*(*t*)^, the daily risk of becoming infected, where v = 1/D is the daily recovery rate (D = 75, [58]), b = 0.55 [59] is the probability of infection given a bite from an infectious mosquito and f(t) is the number of infectious feeders on day t.

Mosquitoes are infected by feeding on an infectious human host with susceptibility determined by their exposure to DBH. For a feeding mosquito, the risk of becoming infected, β_m_(t), is assumed to be related to proportion of infectious humans as follows: $\beta_{m}\left(t\right)=c_{2}\left(1-\frac{c_{1}}{c_{2}I_{H}\left(t\right)+c_{1}}\right)\left(1-h_{s}\right)$, where the risk is discounted by DBH exposure, with *h*_*s*_ = 0.87 for the highest DBH dose. Here, c_1_ = 0.05 controls the initial steepness of the curve and c_2_ = 0.01366 controls the maximum risk, $q=\frac{c_{2}^{2}}{c_{2}+c_{1}}$, which we determine to be 10%, (S3E Fig) [60, 61]. In each potentially infectious compartment, i.e. a feeding compartment occurring at least 12 days after the first feed [9], the number of mosquitoes is multiplied by the probability of being infected during at least one feed occurring at least 12 days ago, giving the number of infectious feeders for that compartment. Summing over all potentially infectious compartments gives the total number of infectious feeders f(t).

We consider interventions in settings with high (85%), moderate (45%), and low (5%) malaria prevalence pre-intervention, mediated by the number of bites per human per mosquito per day (S1 Table). Our quantification of bites per human per mosquito per day is not directly comparable to the standard biting rate parameter, *a*, in the Ross-MacDonald model of malaria transmission [7, 8] as it incorporates both biting rate and mosquito to human ratio.

#### Estimation of DBH efficacy from data

The risk of mating and the risk of *Plasmodium* infection was estimated separately for the DBH-exposed groups and control groups using experimental data (in Fig 1), and the efficacy of the mating and parasite effects were defined to be one minus the relative risk. The mean number of eggs laid was estimated for each group, and the efficacy of the egg effect was defined to be one minus the mean number of eggs in the exposed group, relative to the control. To account for the time-varying nature of the mortality effect, we stratified by day post-exposure. For each day post-exposure, we estimated the daily survival probability with and without DBH from the proportion of mosquitoes that survive to the next day. For the days without observations, we linearly interpolated the number of surviving mosquitoes from the measured time points bracketing the absent observations. For example, if 100 mosquitoes are alive on day 11 and 70 on day 14, we assume that 90 are alive on day 12 and 80 on day 13. See S2 Table for daily mortality values.

The mosquito lifecycle and malaria transmission model was implemented in Python 2.7.6 [62] and analyses were performed in an IPython notebook (version 2.1.0) [63]. Efficacy estimation was done using RStudio (version 0.98) running R (version 3.2.3) [64, 65].

## Supporting Information

S1 Table Model parameters. Symbol, description, standard value used and reference for the parameters found in the model.
(PDF)

S2 Table Daily mortality rates. Daily mortality rates as calculated from the experimental results from Fig 1.
(PDF)

S1 Fig TUNEL/DNA staining of fixed *Anopheles gambiae* ovarioles 24hr after 20E and DBH exposure. Intrathoracic injection of 138 nl of **(A)** 10% ethanol/PBS (injection vehicle control), and **(B)** 38 mM 20E in 10% ethanol/PBS. (**C**) Topical application of 2 μg of DBH in 5% DMSO/acetone (0.4% w/v). The primary (1°F), secondary (2°F), and—where visible—tertiary (3°F) follicles, nurse cells (NC) and the oocyte (OOC) are indicated. Fragmented chromatin (indicating apoptosis) was labelled with FITC (green) using TUNEL. DNA was stained with DAPI (blue). Both 20E injection and DBH exposure induce extensive apoptosis in primary follicles (*) at 24h post treatment. The scale bar (bottom right) represents 50 μm.
(TIF)

S2 Fig Effects of DBH on *P*. *falciparum* infection intensity after topical application. After treatment with 3 DBH doses (2 μg, 0.5 μg, and 0.125 μg), females who developed oocysts following an infectious blood meal showed similar intensity of infection compared to controls. Number of individuals (n) is listed below each DBH condition.
(TIF)

S3 Fig Methodological details of the model. **(A)** The fraction of mosquitoes exposed increases non-linearly with the intervention applied: no effect (gray), DBH alone (pink lines), insecticide alone (blue lines). **(B)** The daily adult survival curve and **(C)** cumulative survival curve as estimated from the experimental data for the model (modeled to reflect Fig 1C, Methods) are shown. For (B)-(C), DBH and insecticide are compared to controls (thick black line) and constant daily mortality (thin black line). **(D)** The daily mortality in the larval stage is a sigmoidal function (solid line) of the larval population size. Previous models [57, 66] have used a linear function (dotted line) for density dependent larval mortality. (**E**) The daily risk of infection by mosquitoes biting infected humans increases quickly at low prevalence in humans and reaches a maximum of 10%.
(TIF)

S4 Fig Effect of indoor spray intervention on the female adult mosquito population and individual age classes. **(A)** The adult mosquito population varies non-linearly under increasing coverage with different DBH doses (pink lines) or insecticide efficacy (blue lines). The vertical yellow bar indicates 85% coverage for which the age distribution of the population is considered in (B). The population size shown is relative to the total female population in the absence of any interventions. **(B)** The age distribution of female mosquitoes in the presence of 2.0 μg DBH (pink) or the absence of any intervention (gray) at 85% coverage, indicated by a yellow bar in (A) and (C). The highlighted days in the x-axis indicate the age range of mosquitoes that are old enough to transmit malaria if infected. **(C)** The potentially infectious adult mosquito population under increasing levels of coverage with different DBH doses (pink lines) or insecticide efficacy (blue lines). This includes females at least 12 days after a blood meal, and in the case of DBH exposure, a proportion of these females are excluded due to reduced *Plasmodium* susceptibility. The yellow bar indicates 85% coverage, for which the age distributions of the population are considered in (B). Without intervention, the proportion of mosquitoes 14 days or older (black line) is 0.22. Insecticide of 60%, 80%, or 100% efficacy and DBH of experimentally determined efficacy (Fig 1) are used.
(TIF)

S5 Fig Density-dependent larval mortality drives variability in the adult population size. **(A)** Increases in egg batch size are associated with increases in egg and larval populations while adult populations show non-linear increases with a peak population size when egg batch size is small. However, larger egg batches are observed in field and experimental settings, likely due to the steep decline in adult population just below the optimal batch size. In particular, due to the dependence on climatic factors, the egg batch size must be substantially larger to avoid population crashes through stochastic drops in egg batch size. **(B)** The older adult mosquito population, i.e. at least 12 days after their first feed, under increasing levels of coverage with different DBH doses (pink lines) or insecticide efficacy (blue lines). The yellow bar indicates 85% coverage, for which the demographics of the population are considered in Fig 3. Without intervention, the proportion of mosquitoes 14 days or older (black line) is 0.22. The increases in older adult population size under the intermediate DBH dose result from decreases in egg batch size leading to a release of density-dependent larval mortality, which is not compensated for by increased adult mortality.
(TIF)

S6 Fig Schematic of human-malaria transmission model. We use a Susceptible-Exposed-Infectious model to track malaria in mosquitoes. Susceptible mosquitoes (S_M_) become exposed (E_M_) by feeding on an infectious human. The force of infection from humans to mosquitoes is a function of the proportion of humans who are infectious (S5E Fig). The latent period in mosquitoes (sporogony) is 12 days, after which the exposed mosquito becomes infectious (I_M_). We use a Susceptible-Infectious model to follow malaria in humans. Susceptible humans (S_H_) become infectious (I_H_) immediately after an infectious mosquito bite. The force of infection from mosquitoes to humans is a function of: (i) the proportion of mosquitoes which are infectious, (ii) the number of bites per mosquito per human per day, and (iii) the per-bite probability of transmission. The mean recovery period for infectious humans is 75 days.
(TIF)

S7 Fig Effect of indoor-spray based interventions on malaria. Effectiveness against malaria considering changes in both coverage (x-axis) and efficacy (line style) as determined from dose response experiments for **(A)** low, **(B)** moderate, or **(C)** high transmission setting. Effectiveness (reduction in malaria prevalence relative to pre-intervention prevalence) at 2 μg DBH (solid pink line) was greater than or comparable to 100% insecticide efficacy, while 0.5 μg (dashed pink line) and 0.125 μg DBH (dotted pink line) had effectiveness similar to that of 80% (dashed blue line) or 60% (dotted blue line) insecticide efficacy, respectively. The maximal DBH efficacy considered is our experimentally determined effects on egg development, mating, mortality, and *Plasmodium* susceptibility (Fig 1).
(TIF)
