# Supplementary material for: Disrupting Mosquito Reproduction and Parasite Development for Malaria Control
Source: PLoS Pathog. 2016 Dec 15;12(12):e1006060. doi: 10.1371/journal.ppat.1006060 (PMC5158081; doi:10.1371/journal.ppat.1006060)
Supplement: S1 Table — Symbol, description, standard value used and reference for the parameters found in the model. (PDF) [file ppat.1006060.s001.pdf]

S1 Table. Model parameters. Symbol, description, standard value used, and reference for the parameters found in the model.

Symbol	Description	Value	Reference
**Structural parameters**			
t_e	Duration of egg stage (days)	3	[1]
t_l	Duration of larval and pupae stages (days)	10	[2]
n_g	Maximum number of gonotrophic cycles	6	[3]
**Lifecycle parameters**			
d_e	Egg mortality (per day)	0.2	text
d_l^{min}	Baseline larva mortality (per day)	0.035	[4]
d_l^{max}	Maximum larva mortality (per day)	0.4	[4]
d_a	Adult mortality without intervention (per day)	varies	S3 Fig, S2 Table
p_m	Probability of mating before feeding	0.5	[5]
n_e	Number of eggs per batch	60	[6]
K	Larval carry capacity	10,000	text
**Malaria parameters**			
S	Length of sporogony (days)	12	[7]
b	Risk of transmission from mosquito to human per infectious bite	0.55	[8]
D	Length of infectious period of humans (days)	75	[9]
N	Bites per mosquito per human per day	$2.15 \cdot 10^{-2}$, $3.78 \cdot 10^{-3}$, $1.09 \cdot 10^{-3}$ #	text
q	Maximum risk of transmission from human to feeding mosquito (per day)	0.1	text
**Intervention parameters**			
h_m	Effective reduction in mating due to DBH	0.25, 0.54, 0.65&	text
h_e	Effective reduction in egg batch size due to DBH	0.26, 0.69, 0.95&	text
h_d	Additional mortality due to DBH	Varies&	S3 Fig, S2 Table
h_s	Effective reduction in *Plasmodium* susceptibility	0.16, 0.56, 0.87&	text
d_c	Additional mortality due to insecticide	0.8	text

#Bites per mosquito per human per day (N) varies with transmission and has three values corresponding to low, moderate and high transmission.

&Intervention parameters (h_m, h_e, h_d, h_s) have three values corresponding to increasing levels of DBH: 0.125 µg DBH, 0.5 µg DBH and 2.0 µg DBH.

1. Yaro AS, Dao A, Adamou A, Crawford JE, Ribeiro JM, Gwadz R, et al. The distribution of hatching time in *Anopheles gambiae*. Malaria journal. 2006;5:19. doi: 10.1186/1475-2875-5-19. PubMed PMID: 16553960; PubMed Central PMCID: PMC1479351.
2. Bayoh MN, Lindsay SW. Temperature-related duration of aquatic stages of the Afrotropical malaria vector mosquito *Anopheles gambiae* in the laboratory. Medical and veterinary entomology. 2004;18(2):174-9. doi: 10.1111/j.0269-283X.2004.00495.x. PubMed PMID: 15189243.
3. Gillies MT, Wilkes TJ. A study of the age-composition of populations of *Anopheles gambiae Giles* and *A. funestus Giles* in North-Eastern Tanzania. Bulletin of entomological research. 1965;56(2):237-62. Epub 1965/12/01. PubMed PMID: 5854754.
4. White MT, Griffin JT, Churcher TS, Ferguson NM, Basanez MG, Ghani AC. Modelling the impact of vector control interventions on *Anopheles gambiae* population dynamics. Parasites & vectors. 2011;4:153. doi: 10.1186/1756-3305-4-153. PubMed PMID: 21798055; PubMed Central PMCID: PMC3158753.
5. Charlwood JD, Pinto J, Sousa CA, Ferreira C, Petrarca V, Rosario Vdo E. 'A mate or a meal'--pre-gravid behaviour of female *Anopheles gambiae* from the islands of Sao Tome and Principe, West Africa. Malaria journal. 2003;2:9. doi: 10.1186/1475-2875-2-9. PubMed PMID: 12801421; PubMed Central PMCID: PMC161788.
6. Dieter KL, Huestis DL, Lehmann T. The effects of oviposition-site deprivation on *Anopheles gambiae* reproduction. Parasites & vectors. 2012;5:235. doi: 10.1186/1756-3305-5-235. PubMed PMID: 23072301; PubMed Central PMCID: PMC3514158.
7. Baton LA, Ranford-Cartwright LC. Spreading the seeds of million-murdering death: metamorphoses of malaria in the mosquito. Trends in parasitology. 2005;21(12):573-80. Epub 2005/10/21. doi: 10.1016/j.pt.2005.09.012. PubMed PMID: 16236552.
8. Smith DL, Drakeley CJ, Chiyaka C, Hay SI. A quantitative analysis of transmission efficiency versus intensity for malaria. Nat Commun. 2010;1:108. doi: 10.1038/ncomms1107. PubMed PMID: 21045826; PubMed Central PMCID: PMCPMC3065713.
9. Collins WE, Jeffery GM. A retrospective examination of mosquito infection on humans infected with *Plasmodium falciparum*. The American journal of tropical medicine and hygiene. 2003;68(3):366-71. PubMed PMID: 12685646.
