# Supplementary material for: Disrupting Mosquito Reproduction and Parasite Development for Malaria Control
Source: PLoS Pathog. 2016 Dec 15;12(12):e1006060. doi: 10.1371/journal.ppat.1006060 (PMC5158081; doi:10.1371/journal.ppat.1006060)
Supplement: S2 Table — Daily mortality rates as calculated from the experimental results from Fig 1. (PDF) [file ppat.1006060.s002.pdf]

S2 Table. Daily mortality rates. Daily mortality rates as calculated from the experimental results from Fig 1.

day	no intervention	0.125 μg DBH	0.5 μg DBH	2.0 μg DBH
0	0.00	0.00	0.00	0.00
1	0.00	0.00	0.00	0.00
2	0.00	0.00	0.00	0.00
3	0.04	0.03	0.04	0.26
4	0.03	0.01	0.04	0.04
5	0.02	0.01	0.04	0.03
6	0.00	0.03	0.00	0.00
7	0.02	0.05	0.02	0.01
8	0.02	0.03	0.05	0.10
9	0.01	0.03	0.10	0.10
10	0.04	0.02	0.05	0.08
11	0.03	0.01	0.04	0.14
12	0.06	0.10	0.11	0.16
13	0.07	0.11	0.12	0.20
14	0.07	0.12	0.14	0.24
15	0.08	0.23	0.16	0.22
16	0.09	0.31	0.20	0.28
17	0.22	0.21	0.20	0.14
18	0.29	0.26	0.25	0.17
19	0.41	0.35	0.33	0.20
20	0.41	0.35	0.33	0.20
21	0.41	0.35	0.33	0.20
22	0.41	0.35	0.33	0.20
23	0.41	0.35	0.33	0.20
24	0.41	0.35	0.33	0.20
25	0.41	0.35	0.33	0.20
26	0.41	0.35	0.33	0.20
