# Supplementary figures and images for: Disrupting Mosquito Reproduction and Parasite Development for Malaria Control
Source: PLoS Pathog. 2016 Dec 15;12(12):e1006060. doi: 10.1371/journal.ppat.1006060 (PMC5158081; doi:10.1371/journal.ppat.1006060)

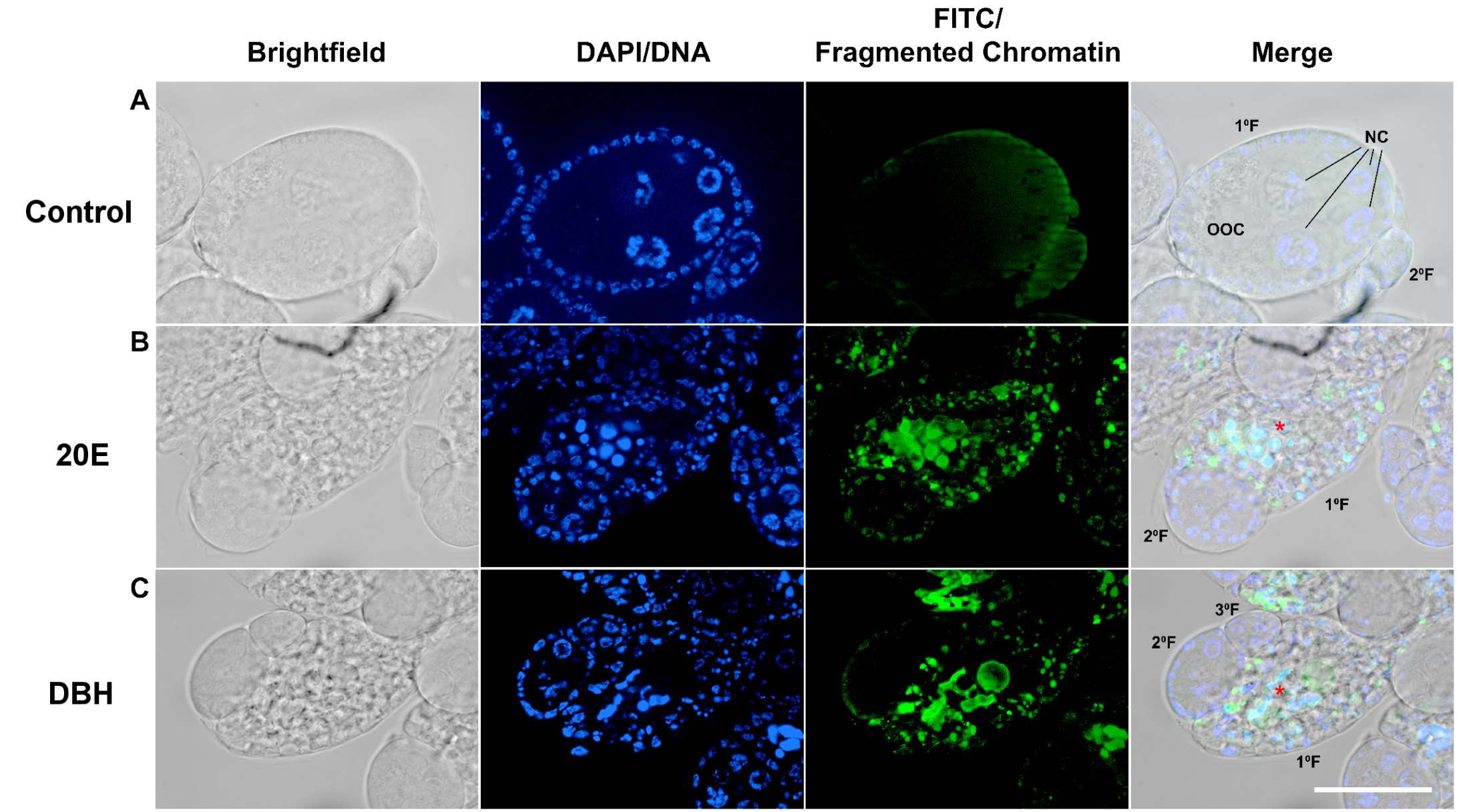

Supplement: S1 Fig — Intrathoracic injection of 138 nl of (A) 10% ethanol/PBS (injection vehicle control), and (B) 38 mM 20E in 10% ethanol/PBS. (C) Topical application of 2 μg of DBH in 5% DMSO/acetone (0.4% w/v). The primary (1°F), secondary (2°F), and—where visible—tertiary (3°F) follicles, nurse cells (NC) and the oocyte (OOC) are indicated. Fragmented chromatin (indicating apoptosis) was labelled with FITC (green) using TUNEL. DNA was stained with DAPI (blue). Both 20E injection and DBH exposure induce extensive apoptosis in primary follicles (*) at 24h post treatment. The scale bar (bottom right) represents 50 μm. (TIF) [file ppat.1006060.s003.tif]

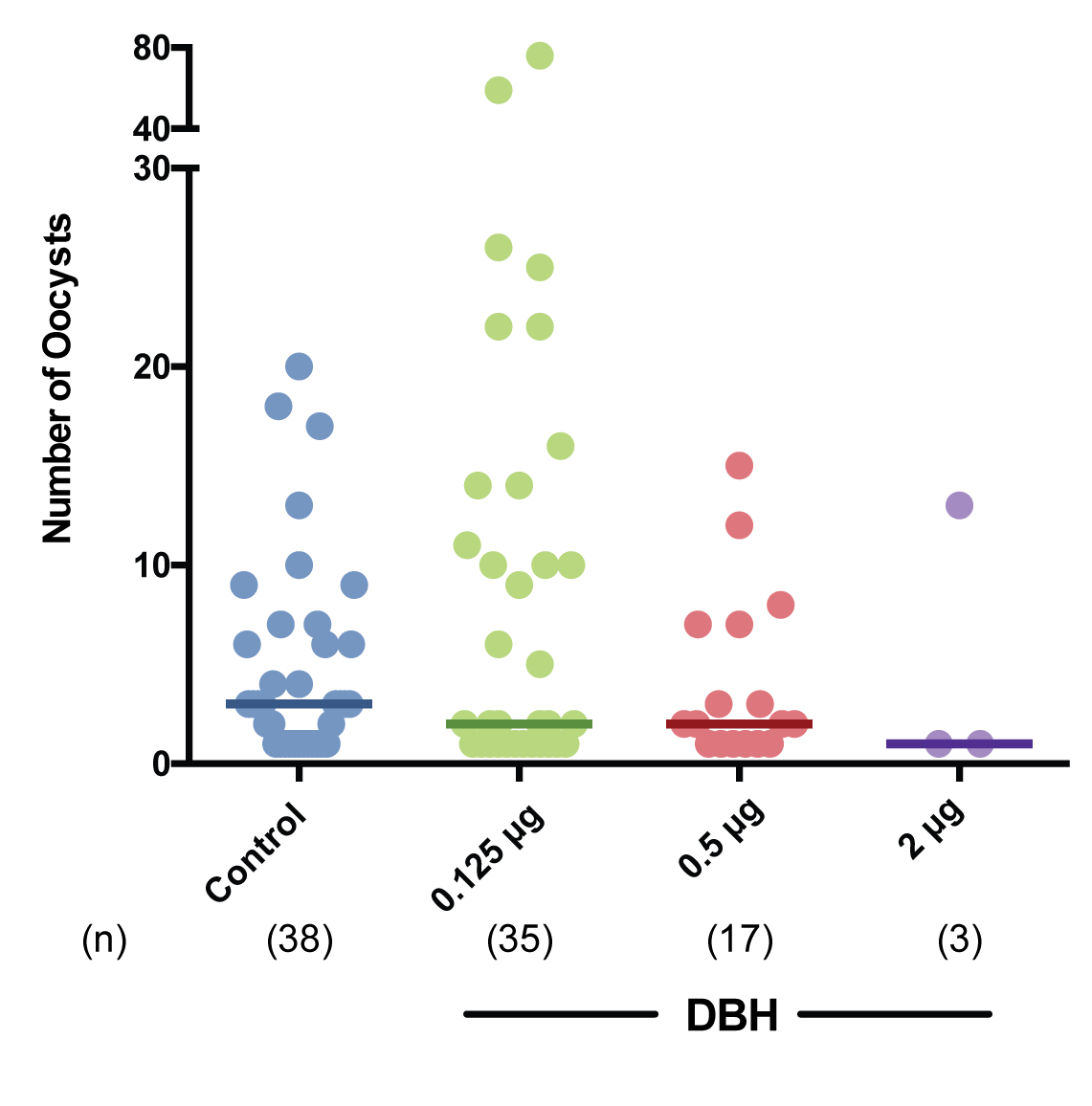

Supplement: S2 Fig — After treatment with 3 DBH doses (2 μg, 0.5 μg, and 0.125 μg), females who developed oocysts following an infectious blood meal showed similar intensity of infection compared to controls. Number of individuals (n) is listed below each DBH condition. (TIF) [file ppat.1006060.s004.tif]

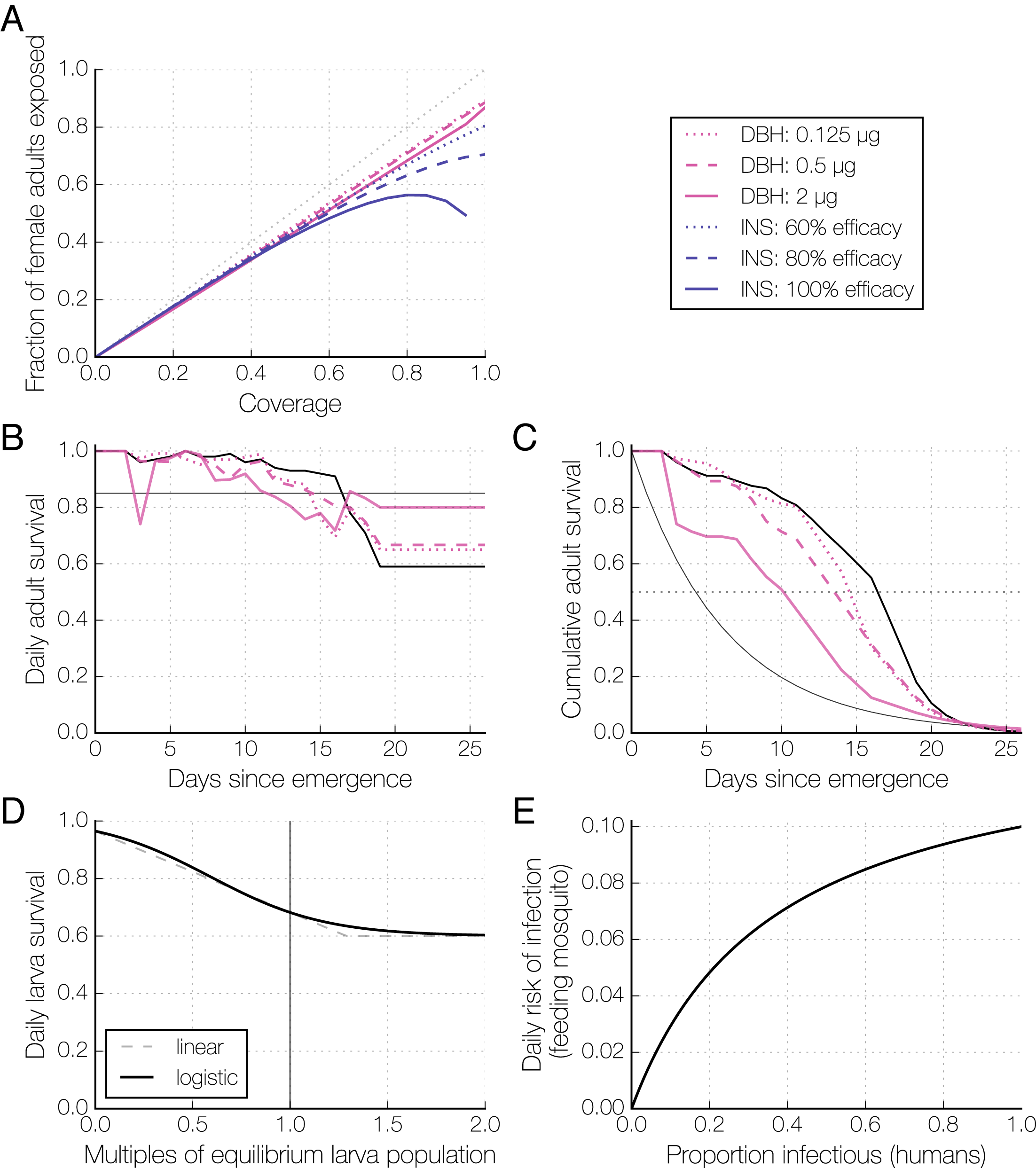

Supplement: S3 Fig — (A) The fraction of mosquitoes exposed increases non-linearly with the intervention applied: no effect (gray), DBH alone (pink lines), insecticide alone (blue lines). (B) The daily adult survival curve and (C) cumulative survival curve as estimated from the experimental data for the model (modeled to reflect Fig 1C, Methods) are shown. For (B)-(C), DBH and insecticide are compared to controls (thick black line) and constant daily mortality (thin black line). (D) The daily mortality in the larval stage is a sigmoidal function (solid line) of the larval population size. Previous models [57, 66] have used a linear function (dotted line) for density dependent larval mortality. (E) The daily risk of infection by mosquitoes biting infected humans increases quickly at low prevalence in humans and reaches a maximum of 10%. (TIF) [file ppat.1006060.s005.tif]

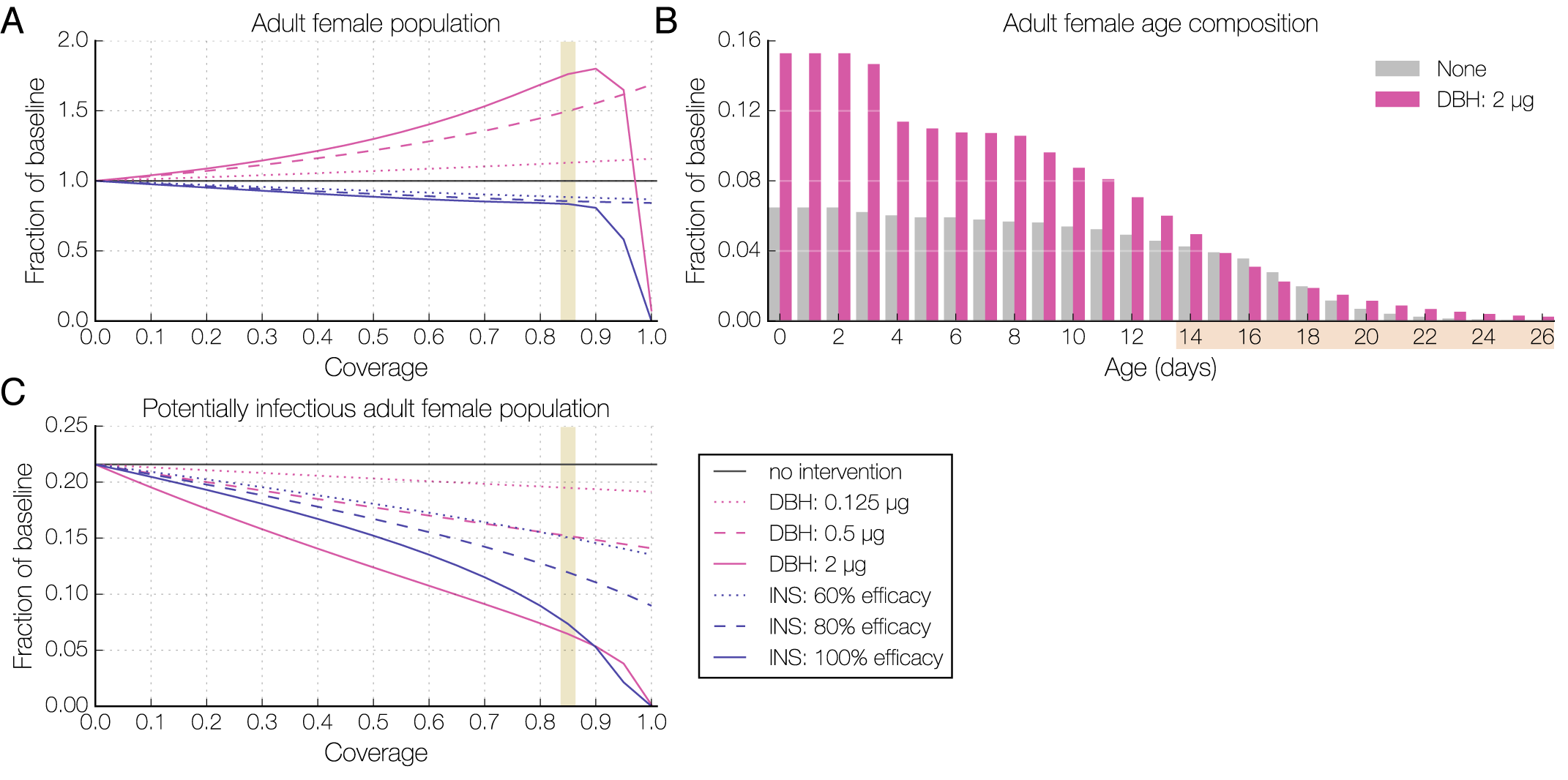

Supplement: S4 Fig — (A) The adult mosquito population varies non-linearly under increasing coverage with different DBH doses (pink lines) or insecticide efficacy (blue lines). The vertical yellow bar indicates 85% coverage for which the age distribution of the population is considered in (B). The population size shown is relative to the total female population in the absence of any interventions. (B) The age distribution of female mosquitoes in the presence of 2.0 μg DBH (pink) or the absence of any intervention (gray) at 85% coverage, indicated by a yellow bar in (A) and (C). The highlighted days in the x-axis indicate the age range of mosquitoes that are old enough to transmit malaria if infected. (C) The potentially infectious adult mosquito population under increasing levels of coverage with different DBH doses (pink lines) or insecticide efficacy (blue lines). This includes females at least 12 days after a blood meal, and in the case of DBH exposure, a proportion of these females are excluded due to reduced Plasmodium susceptibility. The yellow bar indicates 85% coverage, for which the age distributions of the population are considered in (B). Without intervention, the proportion of mosquitoes 14 days or older (black line) is 0.22. Insecticide of 60%, 80%, or 100% efficacy and DBH of experimentally determined efficacy (Fig 1) are used. (TIF) [file ppat.1006060.s006.tif]

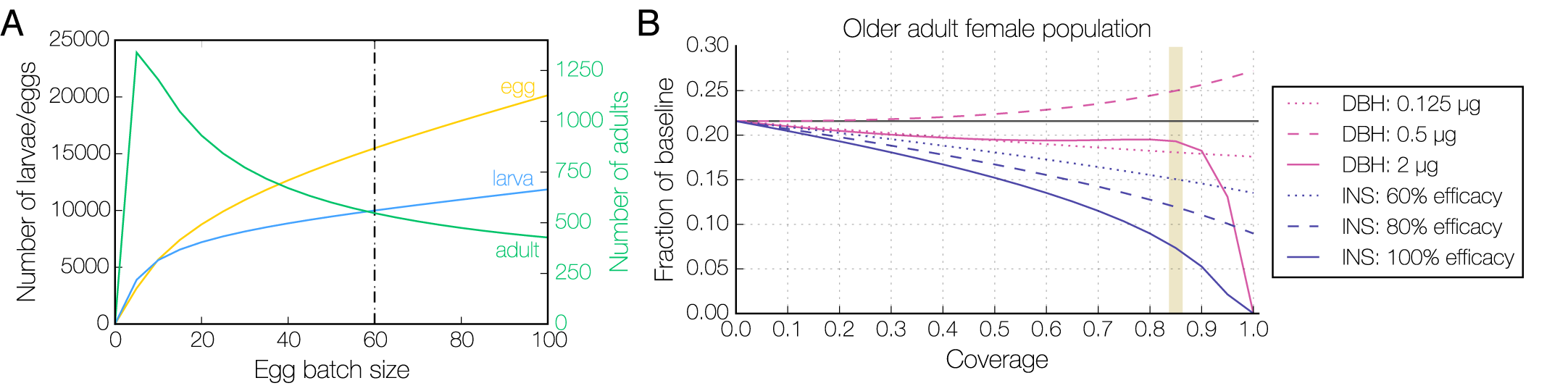

Supplement: S5 Fig — (A) Increases in egg batch size are associated with increases in egg and larval populations while adult populations show non-linear increases with a peak population size when egg batch size is small. However, larger egg batches are observed in field and experimental settings, likely due to the steep decline in adult population just below the optimal batch size. In particular, due to the dependence on climatic factors, the egg batch size must be substantially larger to avoid population crashes through stochastic drops in egg batch size. (B) The older adult mosquito population, i.e. at least 12 days after their first feed, under increasing levels of coverage with different DBH doses (pink lines) or insecticide efficacy (blue lines). The yellow bar indicates 85% coverage, for which the demographics of the population are considered in Fig 3. Without intervention, the proportion of mosquitoes 14 days or older (black line) is 0.22. The increases in older adult population size under the intermediate DBH dose result from decreases in egg batch size leading to a release of density-dependent larval mortality, which is not compensated for by increased adult mortality. (TIF) [file ppat.1006060.s007.tif]

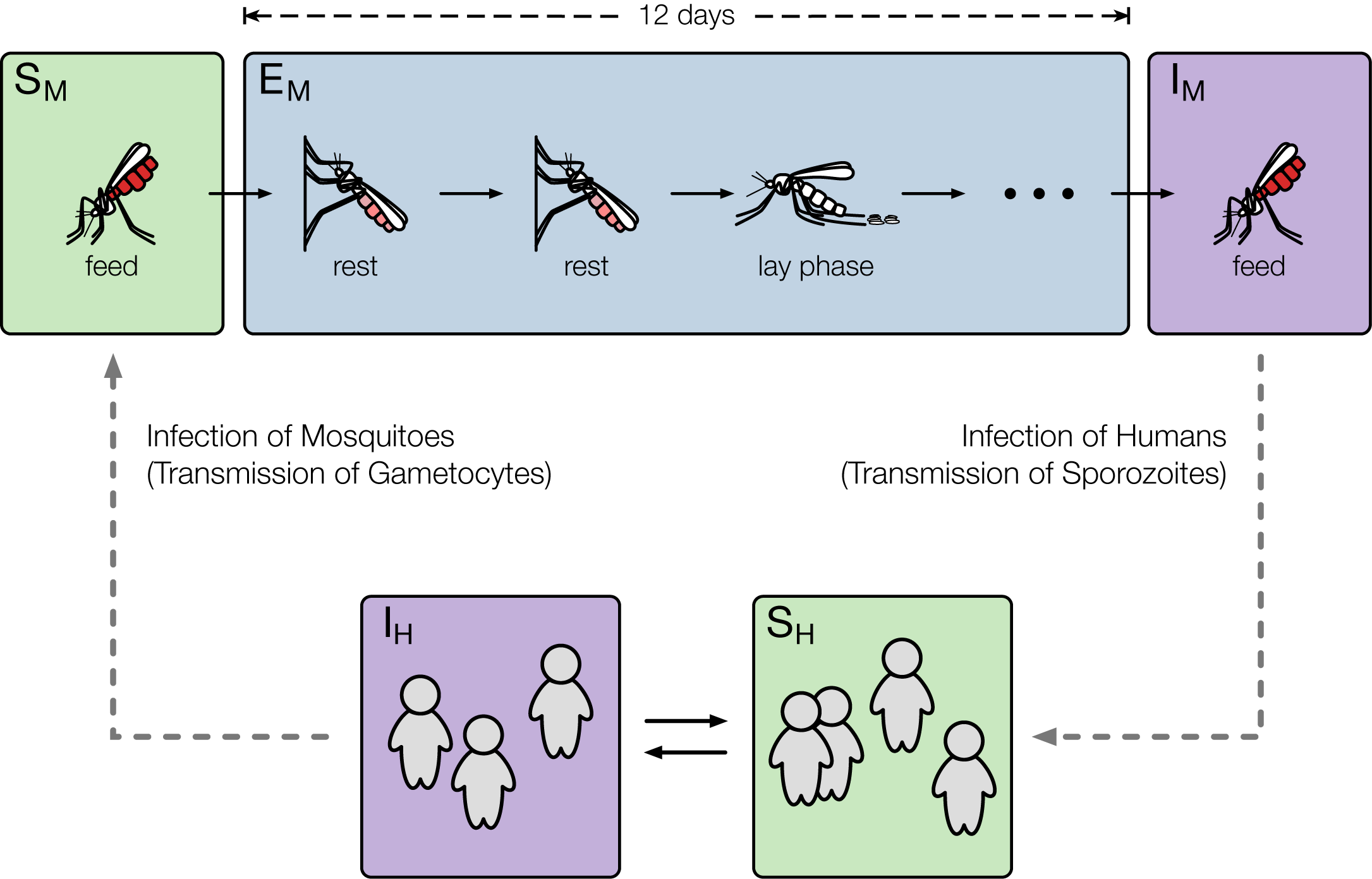

Supplement: S6 Fig — We use a Susceptible-Exposed-Infectious model to track malaria in mosquitoes. Susceptible mosquitoes (SM) become exposed (EM) by feeding on an infectious human. The force of infection from humans to mosquitoes is a function of the proportion of humans who are infectious (S5E Fig). The latent period in mosquitoes (sporogony) is 12 days, after which the exposed mosquito becomes infectious (IM). We use a Susceptible-Infectious model to follow malaria in humans. Susceptible humans (SH) become infectious (IH) immediately after an infectious mosquito bite. The force of infection from mosquitoes to humans is a function of: (i) the proportion of mosquitoes which are infectious, (ii) the number of bites per mosquito per human per day, and (iii) the per-bite probability of transmission. The mean recovery period for infectious humans is 75 days. (TIF) [file ppat.1006060.s008.tif]

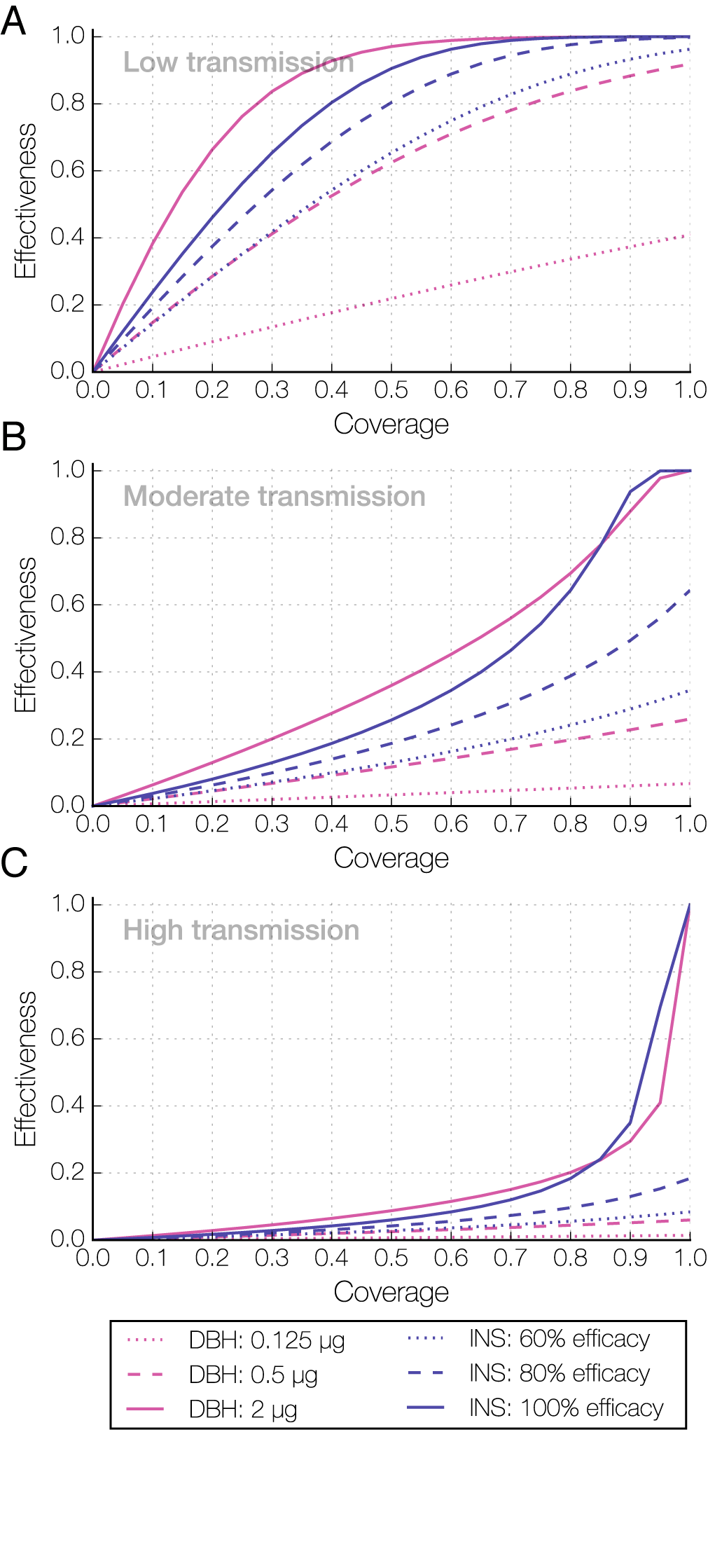

Supplement: S7 Fig — Effectiveness against malaria considering changes in both coverage (x-axis) and efficacy (line style) as determined from dose response experiments for (A) low, (B) moderate, or (C) high transmission setting. Effectiveness (reduction in malaria prevalence relative to pre-intervention prevalence) at 2 μg DBH (solid pink line) was greater than or comparable to 100% insecticide efficacy, while 0.5 μg (dashed pink line) and 0.125 μg DBH (dotted pink line) had effectiveness similar to that of 80% (dashed blue line) or 60% (dotted blue line) insecticide efficacy, respectively. The maximal DBH efficacy considered is our experimentally determined effects on egg development, mating, mortality, and Plasmodium susceptibility (Fig 1). (TIF) [file ppat.1006060.s009.tif]
